# Metabolomics Profiling and In Vitro Genoprotective Effect of *Actinidia chinensis* Planch. var. *deliciosa* (A.Chev.) A.Chev. Leaf Extract

**DOI:** 10.3390/toxics14040324

**Published:** 2026-04-13

**Authors:** Ghanya Al-Naqeb, Mauro Commisso, Sara Boussetta, Rachele De Giuseppe, Hellas Cena

**Affiliations:** 1 Laboratory of Dietetics and Clinical Nutrition, Department of Public Health, Experimental and Forensic Medicine, University of Pavia, 27100 Pavia, Italyrachele.degiuseppe@unipv.it (R.D.G.); hellas.cena@unipv.it (H.C.); 2 Department of Food Sciences and Nutrition, Faculty of Agriculture Food and Environment, University of Sana’a, Sana’a P.O. Box 1247, Yemen; 3 Department of Biotechnology, University of Verona, 37134 Verona, Italy; mauro.commisso@univr.it; 4 Unit of Clinical Nutrition, Istituti Clinici Scientifici Maugeri IRCCS, 27100 Pavia, Italy

**Keywords:** agro-industrial byproduct, *Actinidia deliciosa*, metabolomic profile, cytotoxicity, antigenotoxicity

## Abstract

Leaves of *Actinidia chinensis* Planch. var. *deliciosa* (A.Chev.) A.Chev. (*A. deliciosa*) represent agro-industrial byproducts with potential for valorization. The present study evaluated the metabolomics profiling, cytotoxicity, genotoxicity, and antigenotoxicity of the methanolic extract of *A. deliciosa* leaves. The metabolomics profiling was determined using an untargeted metabolomic approach employing UPLC-HRMS. Cytotoxicity, genotoxicity, and antigenotoxicity were assessed in Chinese hamster ovary K1 (CHO-K1) cells using the in vitro cytokinesis-block micronucleus (CBMN) assay. The metabolic profile of *A. deliciosa* leaf extracts revealed the presence of three major classes of secondary/specialized metabolites: proanthocyanidins, flavonols, and triterpenoid saponins. Medium-polar metabolites were monomeric fla-van-3-ols, such as (+)-catechin and (−)-epicatechin, oligomeric procyanidins and prodelphinidins, and flavonols. Certain glycosylated flavonols and their derivatives, such as myricetin, quercetin, and kaempferol. Low-polarity metabolites were characterized by low-polarity triterpenoids such as maslinic, corosolic, oleanolic, and ursolic acids. At concentrations of 37.5, 75, and 150 µg/mL, the extract did not significantly increase micronuclei frequency compared to untreated control cells, indicating an absence of genotoxic potential. Moreover, co-treatment of CHO-K1 cells with the extract and mitomycin C (MMC) at 0.025 µg/mL resulted in a significant reduction in micronuclei formation induced by MMC at concentrations of 75 and 150 µg/mL, suggesting antigenotoxic activity likely associated with the phytochemical constituents presented in the extract.

## 1. Introduction

The *Actinidia* genus, belonging to the Actinidiaceae family, comprises more than 70 species and is widely distributed across various regions of the world [1]. Plants from this genus have been traditionally utilized for their medicinal properties, offering symptomatic relief for gastrointestinal disorders, dyspepsia, rheumatism, and hemorrhoids, as well as serving as complementary therapy in cancer treatment [2]. Among the species, *Actinidia chinensis* Planch. var. *deliciosa* (A.Chev.), A.Chev. (*A. deliciosa*) is notable for generating substantial quantities of residues and waste throughout its production cycle [3]. These residues, comprising skins, seeds, leaves, shoots, flowers, and roots, are rich in bioactive compounds [3,4]. The effective valorization of these residues presents both a challenge and an opportunity for their integration into the food, pharmaceutical, and cosmetic industries [3].

Leaves of *A. deliciosa* represent a significant agro-industrial byproduct with potential for valorization due to their rich content of bioactive compounds. Previous studies have reported the extraction of various phenolic compounds from *A. deliciosa* leaves, with quercitrin and rutin identified as the major constituents [5]. Additional compounds such as proanthocyanidins, quinic acid, myricitrin, quercetin, kaempferol, and triterpene acid-O-hexoside were also detected [5,6]. *A. deliciosa* leaves phenolic-rich extracts have demonstrated notable biological activities in vitro, including modulation of cellular protein expression and enzymatic functions [7]. Moreover, methanolic extracts of *A. deliciosa* leaves have been shown to suppress postprandial blood glucose elevation in mice, and the effect is attributed to the inhibition of α-amylase and α-glucosidase activities by phytochemical constituents presented in the extract [8]. These findings suggest that *A. deliciosa* leaves may serve as a novel resource for the prevention of metabolic disorders, such as diabetes.

Methanolic extracts derived from freeze-dried *A. deliciosa* fruits have previously been shown to exhibit neither genotoxic nor cytotoxic effects on human peripheral blood lymphocytes [9]. Furthermore, these fruit extracts demonstrated radioprotective properties at concentrations of 400 and 800 µg/mL, significantly reducing chromosomal aberration frequencies following irradiation exposure [9]. In addition, consumption of *A. deliciosa* juice has been associated with DNA protection against oxidative damage, as demonstrated in both single-dose human studies and long-term supplementation trials using the comet assay [10]. Despite these promising findings, data on the genotoxic or antigenotoxic potential of *A. deliciosa* leaf extracts remain lacking in the scientific literature. However, *A. deliciosa* leaf extracts have exhibited phytotoxic effects, including inhibition of radicle growth in *Lactuca sativa* L. [11]. Notably, (−)-epicatechin has been identified as one of the phytotoxic compounds in *A. deliciosa* leaves [12].

As part of the Italian National Biodiversity Center’s initiative [13], in bioprospecting and bioactivity, this research aims to enhance plant-based resources by identifying bioactive secondary metabolites for innovative applications across industries, including food, pharmaceuticals, cosmetics, and materials. Specifically, we characterized the metabolomic profile and evaluated the genotoxic and antigenotoxic properties of Italian medicinal flora, with a particular focus on *A. deliciosa* leaf extracts. This study represents the first assessment of the genotoxicity and antigenotoxicity of *A. deliciosa* leaf extracts, providing essential preliminary data for their safety evaluation and potential future applications. To this end, we employed the cytokinesis-block micronucleus (CBMN) assay to assess genotoxicity and cytotoxicity in the Chinese hamster ovary K1 (CHO-K1) cell line, a well-established rodent mammalian cell model. An automated CBMN assay was conducted using image analysis via widefield fluorescence microscopy to ensure objective and high-throughput scoring of nuclear anomalies. Our results indicate that the methanolic extract of *A. deliciosa* leaves is rich in diverse bioactive metabolites, does not induce genotoxic effects in CHO-K1 cells, and exhibits genoprotective potential against genotoxic agents under the tested conditions. These findings support the safety profile of *A. deliciosa* leaves extract and highlight its potential as a source of bioactive compounds for health-promoting applications.

## 2. Materials and Methods

### 2.1. Chemicals

LC–MS-grade methanol was purchased from Honeywell (Seelze, Germany), while MMC and cytochalasin B were provided by D.B.A. Italia S.r.l. (Milan, Italy). BioSigma (USA) supplied the 3-(4,5-dimethylthiazol-2-yl)-2,5-diphenyltetrazolium bromide (MTT). Hoechst 33342 Staining Dye Solution was sourced from Abcam via Prodotti Gianni S.r.l. (Milan, Italy). All other cell culture reagents, including media, supplements, consumables, dimethyl sulfoxide (DMSO; 99.9%), and formaldehyde, were obtained from Euroclone S.p.A. (Milan, Italy).

### 2.2. Plant Collection and Extraction

Leaves of *A. deliciosa* were collected on 27 October 2022 from nine fruit-bearing plants located in the orchard of a local producer in Verona, Italy. Sampling was performed in triplicate, and each biological replicate consisted of leaf material pooled from three randomly chosen and independent trees. Immediately after harvesting, leaf samples were frozen in liquid nitrogen, ground to a fine powder using an A11 basic analytical mill (IKA-Werke, Staufen, Germany), and stored at −80 °C until extraction. Approximately 1 g of frozen powdered tissue was extracted with 10 volumes (*w*/*v*) of LC–MS-grade methanol. The suspensions were vortex-mixed for 30 s, sonicated for 10 min in an ice-cooled ultrasonic bath operating at 40 kHz (SOLTEC, Milan, Italy), and centrifuged at 14,000× *g* for 10 min at 4 °C. The supernatants were collected, subdivided into aliquots of 1 mL, and kept at −20 °C prior to LC–MS analysis. Selected aliquots were completely evaporated to dryness using a SpeedVac concentrator (Heto-Holten, Frederiksborg, Denmark) for subsequent analyses.

### 2.3. Metabolomics Profiling

For metabolomics profiling, methanol extracts were diluted 1:50 with LC–MS-grade water (Honeywell, Seelze, Germany) and filtered through 0.22 μm Minisart filters (Sartorius-Stedim Biotech, Göttingen, Germany). Chromatographic separation and untargeted metabolomics analyses were carried out as previously described [14]. Briefly, the chromatographic gradient started with 1% solvent B held for 1 min, linearly increased to 40% at 10 min, 70% at 13.5 min, 90% at 15 min, and 99% at 16.5 min, maintained for 3.5 min before returning to 1% at 20.1 min and held isocratically until 25 min. The UPLC–HRMS analyses were carried out with an Xevo G3 QqTOF mass spectrometer (Waters, Milford, MA, USA) equipped with an ESI ion source performing both positive and negative ionization modes, and by using injection volumes of 1 μL and 5 μL, respectively. In addition, a FAST-DDA method was employed in both modes to enhance metabolite annotation. Tentative metabolite identification was based on accurate mass measurements, retention times, and MS/MS fragmentation patterns, which were cross-referenced against an internal library of authentic standards and public databases including MassBank, HMDB, PubChem, and MoNA. The levels of metabolite identification were assigned according to the criteria proposed by the Metabolomics Standards Initiative [15], with level 1: identified compounds, level 2: putatively annotated compounds, and level 3: putatively characterized compound classes.

### 2.4. Cytotoxicity Assessment

Cytotoxicity and genotoxicity of *A. deliciosa* leaf methanolic extract were assessed using the CHO-K1 cell line. This cell line is widely employed in toxicity studies owing to its rapid proliferation and relatively stable karyotype (22 ± 2 chromosomes) [16,17]. Moreover, CHO-K1 cells have shown high sensitivity (79%) to known carcinogenic compounds in previous studies [18]. The CHO-K1 cell line (603480) was obtained from CLS Cell Lines Service (GmbH, Germany). Cells were maintained in Ham’s F12 medium supplemented with 10% fetal bovine serum (FBS), 1% penicillin/streptomycin, and 1% glutamine. A stock solution of *A. deliciosa* methanolic extract was prepared in dimethyl sulfoxide (DMSO) at a concentration of 100 mg/mL. This stock was serially diluted in culture medium to obtain seven two-fold dilutions ranging from 500 µg/mL to 9.4 µg/mL. Each concentration was tested in triplicate, and all experiments were independently repeated three times. To ensure sterility, all procedures were carried out under a biosafety cabinet.

The cytotoxicity of the *A. deliciosa* methanolic extracts was evaluated using the MTT (3-(4,5-dimethylthiazol-2-yl)-2,5-diphenyltetrazolium bromide) assay, based on the protocol described [19]. CHO-K1 cells were seeded into 96-well flat-bottom microplates (Primo^®^ Multiwall plates, Euroclone S.p.A., Italy) at a density of 3000 cells/well and allowed to adhere for 24 h at 37 °C in a 5% CO_2_ atmosphere. Following the initial incubation, cells were exposed for 24 h to varying concentrations of *A. deliciosa* extract. Experimental controls included a vehicle control (0.4% DMSO) and a negative control (cell culture medium only). After the treatment period, the medium was replaced with 20 µL of MTT solution (5 mg/mL in PBS) per well, and the plates were incubated for an additional 4 h under the same conditions at 37 °C in a 5% CO_2_ atmosphere. The medium was then carefully removed, and 100 µL of 100% DMSO was added to each well to dissolve the resulting formazan crystals. The absorbance was measured at a primary wavelength of 570 nm, with a reference wavelength of 690 nm for background correction, using a microplate reader (Synergy). Cell viability was subsequently calculated as a percentage relative to the control wells according to the formula provided in Equation (1):

(1) $$Viability\%\;=100\times \frac{\left[\left(number\;of\;treated\;cells\right)-\;\left(number\;of\;umber\;of\;untreated\;cells\right)\right]}{number\;of\;untreated\;cells}$$

### 2.5. Genotoxic and Antigenotoxic Study

The in vitro micronucleus assessment was performed using the CBMN assay, following established protocols [19], and in compliance with OECD Test Guideline 487 [20]. CHO-K1 cells were seeded in 96-well plates at a density of 1000 cells/well and maintained for 24 h in a humidified atmosphere (37 °C, 5% CO_2_). After the initial pre-incubation, cells were treated with a negative control 0.3% DMSO), a positive control mitomycin C (MMC) at 0.025 µg/mL, or *A. deliciosa* extract at three concentrations (37.5, 75, 150 µg/mL), both alone and in combination with MMC. Following a 24 h incubation, the cells were washed with PBS and replenished with fresh medium containing 3 µg/mL cytochalasin B to arrest cytokinesis. After another 24 h, the cells were fixed in 4% formaldehyde for 15 min, rinsed with PBS, and stained with Hoechst 33342 100 µL/well) for 30 min at room temperature under light-protected conditions.

To determine the appropriate concentration range for *A. deliciosa* extract, an MTT assay was performed in accordance with OECD cytotoxicity guidelines (threshold of 55 ± 5%). Since concentrations above 150 µg/mL resulted in more than 50% cytotoxicity in CHO-K1 cells, 150 µg/mL was established as the maximum test dose, while 37.5 µg/mL was defined as the non-cytotoxic minimum. Experiments were conducted in triplicate across three independent trials, with at least 1000–2000 binucleated cells analyzed per concentration in each run.

### 2.6. Evaluation of Cytotoxicity Within the CBMN Assay

Cytotoxicity was assessed as an integral component of the CBMN assay to ensure valid genotoxicity data, adhering to current regulatory recommendations. OECD guidelines [20] stipulate that the highest test concentration should not induce cytotoxicity exceeding 55% ± 5% to avoid irrelevant positive results or compromise cell division kinetics. The assessment was performed on the same CHO-K1 cells used for micronucleus induction analysis, and three concentrations of *A. deliciosa* extract, selected based on preliminary MTT assay results, were evaluated. Cytotoxicity was determined using Cytokinesis-Block Proliferation Index (CBPI as previously described [21]. The CBPI reflects the average number of cell cycles completed during exposure to cytochalasin B and is inversely related to the proportion of binucleated versus mononucleated cells. Cytotoxicity percentages were determined using the calculations outlined in Equations (2) and (3):

(2) $$CBPI=100\times \frac{\left[N_{1}\;+\;2\times N_{2}\;+\;3\times N_{3}\right]\;\;\;\;\;\;\;\;\;\;\;\;\;\;\;\;\;\;}{\;\;total\;number\;of\;cells}$$

(3) $$\%\;Cytotoxicity\;\left(CBPI\right)=100-\left[100\times \frac{CBPI\;of\;treated\;cells-1}{CBPI\;of\;untreated\;cells-1}\;\right]\;$$

In this context, *N*_1_ corresponds to the count of mononucleated cells, *N*_2_ to binucleated cells, and *N*_3_ to multinucleated cells.

### 2.7. Fluorescence Microscopy

DAPI-stained cell images were acquired using a Leica DMi8S inverted widefield microscope (Wetzlar, Germany) integrated with LAS X software (https://www.leica-microsystems.com/products/microscope-software/p/leica-las-x-ls/downloads/ (accessed on 23 January 2026). The imaging system featured a Leica DFC 9000 GT CMOS camera and an X-CITE 200DC light source. For the acquisition, a 40× dry objective (Leica HC PL Fluotar L 40×/0.60) was used to capture an array of 64 adjacent fields of view per experimental condition. The DAPI signal was excited and detected using the corresponding filter set (excitation 340–380 nm, dichroic mirror 400 nm, emission LP 425 nm). The experiment included three technical replicates (wells) for each concentration and was biologically repeated three times. In each run, the goal was to score more than 1000 binucleated cells per concentration. All captured images were numerically coded and stored systematically.

### 2.8. Detection of Micronuclei Using CellProfiler Software

The micronucleus assay can be accelerated and made more reproducible through automated image-based analysis. In this study, CellProfiler (version 4.2.6), an open-source software developed by the Broad Institute [22], was employed to automatically identify binucleated cells and micronuclei. Prior to analysis, Leica Lightning adaptive deconvolution was applied to improve the signal-to-noise ratio and recover spatial detail. The analysis pipeline, adapted from our previous work [21], comprised three major stages. First, primary objects (nuclei) were detected, segmented, and classified as mononucleated, binucleated, or polynucleated based on morphological features, with nuclear clusters excluded. Second, secondary and tertiary objects (cells and cytoplasm) were defined, with cytoplasmic regions generated by expanding cellular boundaries and subtracting nuclear areas. Finally, micronuclei were identified according to size and shape criteria and assigned to their corresponding parent nuclei in mono- or binucleated cells [21].

### 2.9. Statistical Analysis

Statistical results were analyzed with GraphPad Prism 7.0, where data are presented as the mean ± standard deviation (SD). To evaluate differences between groups, a one-way ANOVA was utilized, followed by Tukey’s post hoc test for multiple comparisons. We defined statistical significance at a threshold of *p* < 0.05, with *p* < 0.01 denoting high statistical significance.

## 3. Results

### 3.1. Specialized Metabolites in A. deliciosa Leaves

**Figure 1:**
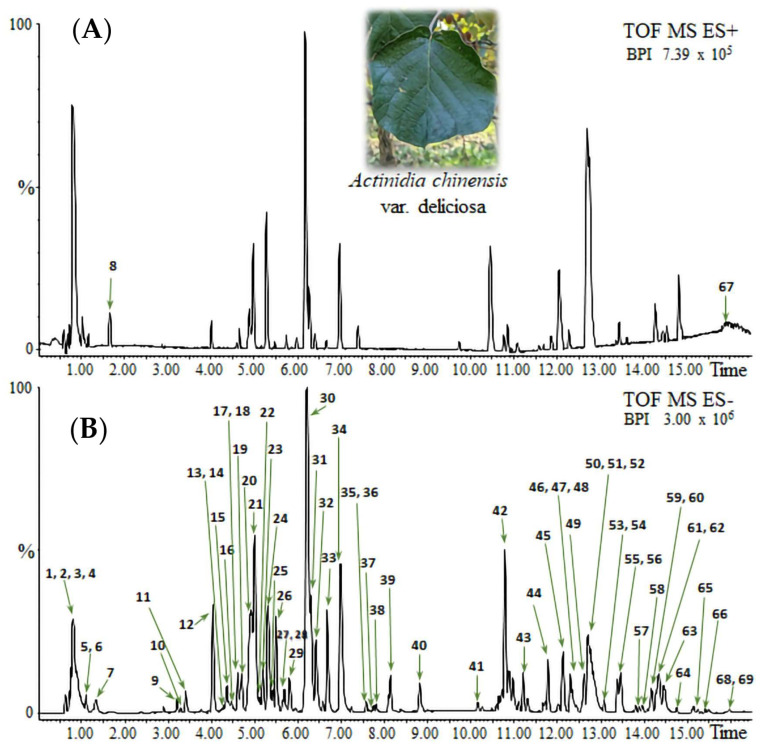
UPLC–HRMS chromatograms of *A. deliciosa* leaf methanol extracts: (**A**) positive and (**B**) negative ionization modes.

The UPLC–HRMS analyses were performed by following an untargeted metabolomics approach on methanol extracts of *A. deliciosa* leaves. The chromatographic profiles were obtained in both positive and negative ionization modes, as illustrated in Figure 1 and Table 1. The tentatively assigned compound identities and the corresponding chromatographic peaks are summarized in Supplementary Table S1. Additional details, including retention times, experimental *m*/*z* values, molecular formulas, and diagnostic fragment ions, are also reported in Supplementary Table S1.

The metabolic profile of *A. deliciosa* leaf extracts revealed the presence of three major classes of secondary/specialized metabolites: proanthocyanidins and flavonols, both belonging to the phenolic compound group, and triterpenoid saponins. The initial chromatographic region (0–5 min) was mainly characterized by primary metabolites, including di-, tri-, and tetrahexoses (peaks 1–4), citric acid (peak 7), and vitamin-related molecules such as ascorbic acid hexoside (peak 5) and pyridoxine hexoside (peak 8). Medium-polar metabolites eluted in the middle region of the chromatograms (5–15 min). In this portion, the most abundant metabolites were monomeric flavan-3-ols, such as (+)-catechin (peak 18) and (−)-epicatechin (peak 24), oligomeric procyanidins and prodelphinidins, ranging from dimers to tetramers (peaks 10–27), and flavonols. Certain glycosylated flavonols, such as myricetin, quercetin, and kaempferol, were unambiguously identified. The most abundant among them were quercetin-3-O-rutinoside (rutin, peak 30), myricetin-3-O-rhamnoside (myricitrin, peak 31), quercetin-3-O-glucoside (isoquercetin, peak 32), kaempferol-3-O-rutinoside (nicotiflorin, peak 33), and kaempferol-3-O-glucoside (astragalin, peak 34). Additionally, other flavonol derivatives containing mono- and disaccharidic residues were detected. Phlorizin (peak 36), detected as a formic acid adduct, was also present as a representative dihydrochalcone glycoside.

Compounds eluting after 18 min were largely composed of low-polar pentacyclic triterpenoids, especially ursane and oleanane-type triterpenic saponins (peaks 37–68), including hydroxylated, acetylated, and coumaroylated derivatives. Maslinic acid (peak 62), corosolic acid (peak 63), oleanolic acid (peak 68), and ursolic acid (peak 69) were unambiguously identified. Additionally, actinidic acid and its structural isomers, which were previously identified in *Actidinia* [23], were detected and putatively confirmed by accurate-mass and MS/MS data comparison with spectral databases. Towards the end of the chromatographic elution, several glycerolipid species were observed, such as digalactosyl and monogalactosyl monoacylglycerols (C18:3; peaks 53 and 57), and one carotenoid, violaxanthin or a structural isomer (peak 67). A few minor and currently unassigned compounds (peaks 17, 25, 42–44) were consistently detected across replicates but could not be confidently annotated.

### 3.2. Cytotoxicity Assessment and Concentration Determination

**Figure 2:**
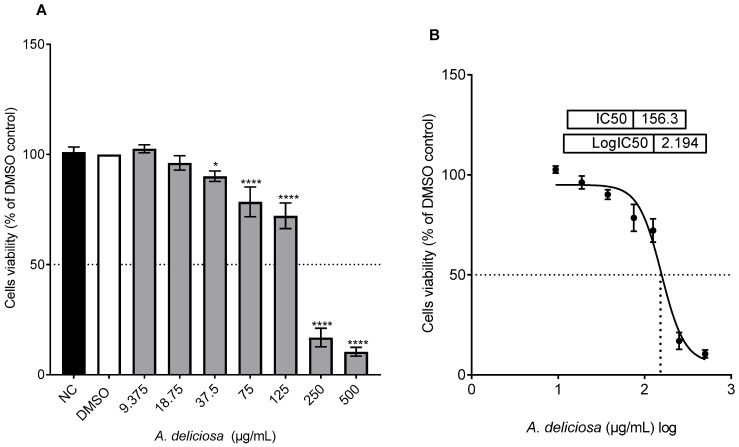
(**A**) Impact of different concentrations of *A. deliciosa* extract (0–500 µg/mL) on CHO-K1 cell viability, expressed as a percentage relative to the DMSO control, after 24 h of treatment, as determined by the MTT assay. Data are shown as mean ± standard deviation from three independent experiments. Statistical significance was assessed using ANOVA followed by the Tukey multiple comparison post-test (* *p* <0.05, **** *p* < 0.0001). (**B**) Nonlinear regression analysis of log-transformed concentrations.

The in vitro cytotoxicity of the crude *A. deliciosa* extract against CHO-K1 cells was assessed using the MTT cell viability assay (Figure 2A,B). After 24 h of exposure, extract concentrations below 37.5 μg/mL produced no statistically significant reduction in cell viability. However, concentrations above this threshold elicited a marked, dose-dependent cytotoxic effect (37.5–500 μg/mL). At the highest concentration tested (500 μg/mL), cell viability dropped to less than 10% relative to the DMSO-treated control. The calculated IC_50_ value for the methanolic leaf extract of *A. deliciosa* was 156 μg/mL.

### 3.3. Genotoxicity Assessment

#### 3.3.1. Cytotoxicity Evaluation Under the CBMN Assay

**Figure 3:**
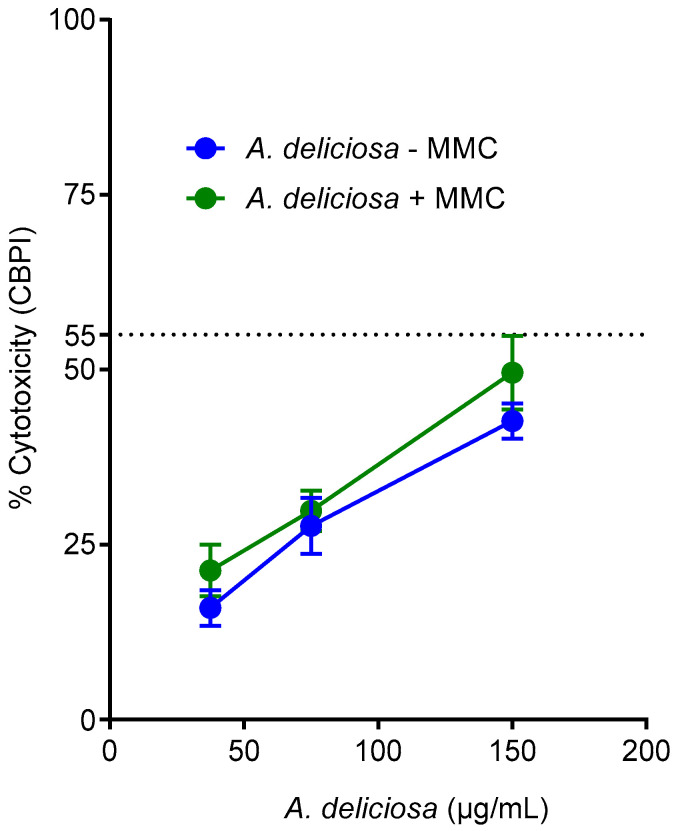
Cytotoxicity of *A. deliciosa* extracts in CHO-K1 cells during the CBMN assay, both independently and in combination with Mitomycin C (MMC). Data points represent the mean values derived from three independent experiments.

Three concentrations of *A. deliciosa* extract (37.5, 75, and 150 µg/mL) were selected based on the IC_50_ value of 156 µg/mL determined by the MTT assay. The highest concentration tested (150 µg/mL) induced cytotoxicity below the 55 ± 5% threshold relative to untreated controls. Cytotoxicity was assessed alongside genotoxicity in the CBMN assay using the CBPI method [21]. CBPI analysis demonstrated a dose-dependent increase in cytotoxicity with *A. deliciosa* extract, yielding 15.94 ± 1.79%, 27.66 ± 1.73%, and 42.64 ± 2.06% at 37.5, 75, and 150 µg/mL, respectively (Figure 3, blue line). Based on these results, 150 µg/mL was selected as the maximum concentration for subsequent micronucleus assays. Co-treatment with *A. deliciosa* extract and MMC further enhanced cytotoxicity compared to MMC alone (14.25 ± 2.17%). In combination, cytotoxicity increased to 21.30 ± 0.82%, 29.83 ± 5.56%, and 49.69 ± 0.53% at 37.5, 75, and 150 µg/mL, respectively, relative to MMC alone (8.66 ± 2.25%) (Figure 3, green line).

At the highest concentration (150 μg/mL), the combination of the extract and MMC exhibited greater cytotoxicity (49.69 ± 0.53%), compared to the extract alone (42.64 ± 2.06%). This sub-additive effect may be attributed to the intrinsic genotoxic action of MMC, which induces cell cycle arrest [24].

#### 3.3.2. Genotoxicity Assessment of *A. deliciosa* Leaves Extract

**Figure 4:**
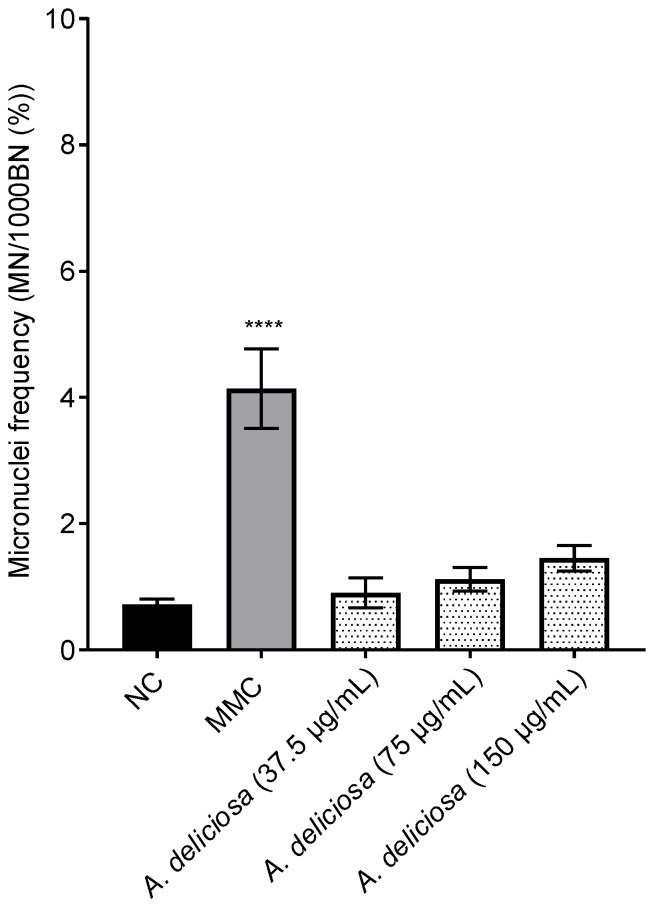
Micronucleus induction in CHO-K1 cells following treatment with *A. deliciosa* extract. Cells were exposed to varying concentrations of the extract (37.5, 75, and 150 μg/mL), a negative control (NC), or a positive control (mitomycin C, 00.025 μg/mL) for 24 h, followed by 24 h incubation with 3 μg/mL cytochalasin B. Micronuclei frequency (%) = binucleated cells with MN/binucleated cells *100. Results are expressed as the mean ± standard deviation from three independent experiments. Statistical analysis was performed via one-way ANOVA and Tukey’s post hoc test; significance is indicated as **** *p* < 0.0001 versus NC.

**Figure 5:**
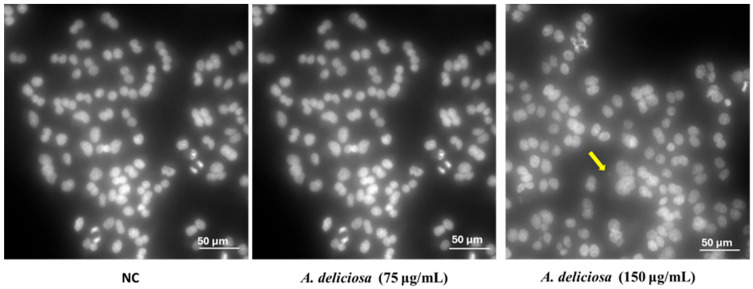
Micronuclei formation in binucleated CHO-K1 cells after non-treatment (NC) or treatment with *A. deliciosa* extract at 75 μg/mL and 150 μg/mL. Morphological observations in the CHO-K1 cells were observed using DAPI staining (Hoechst 33342) under a fluorescence microscope at 40× magnification. Micronuclei are indicated by yellow arrow; the white line represents a scale bar of 50 µm.

The genotoxic potential of *A. deliciosa* leaf extract was assessed using the CBMN assay, a widely used and sensitive method for evaluating genotoxicity in both in vitro and in vivo systems. To validate the assay’s sensitivity, MMC at a concentration of 0.025 μg/mL served as a positive control. As anticipated, MMC significantly increased the frequency of micronuclei compared to untreated control cells (NC), confirming the assay’s capability to detect genotoxic effects. The *A. deliciosa* leaf extract was tested at concentrations of 37.5, 75, and 150 μg/mL. Treatments at all concentrations did not induce statistically significant increases in micronucleus frequency compared to the NC (Figure 4), indicating that, under the conditions tested, the extract is nongenotoxic to CHO-K1 cells. Representative fluorescence microscopic observation of micronuclei in cytokinesis-blocked CHO-K1 cells stained with DAPI is shown in Figure 5.

#### 3.3.3. Antigenotoxic Protection by *A. deliciosa* Extract

**Figure 6:**
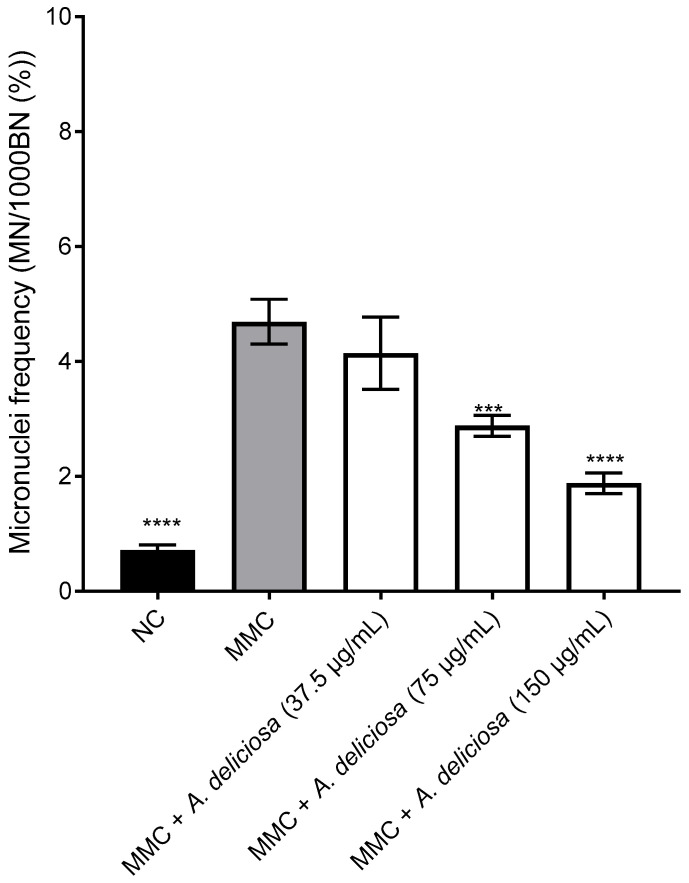
Inhibitory effect of *A. deliciosa* extract on the genotoxicity induced by MMC in CHO-K1 cells. Cells were either untreated (NC), treated with the positive control MMC (0.025 μg/mL), or co-treated with MMC and three concentrations of *A. deliciosa* extract (37.5, 75, and 150 μg/mL) for 24 h for 24 h. This was followed by a 24 h incubation with 3 μg/mL cytochalasin B. Data are presented as mean ± standard deviation from three independent experiments. Statistical significance was determined using one-way ANOVA followed by Tukey’s multiple comparisons test (GraphPad Prism 7). *** *p* < 0.001 and **** *p* < 0.0001 denote statistical significance compared to the MMC-treated positive control.

**Figure 7:**
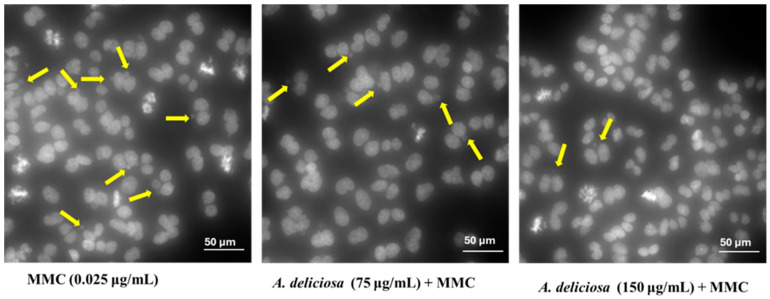
Images illustrating micronuclei formation in binucleated CHO-K1 cells after treatment with MMC alone or in combination with the *A. deliciosa* extract at 75 μg/mL and 150 μg/mL. Morphological observations in the CHO-K1 cells were observed using DAPI staining (Hoechst 33342) under a fluorescence microscope at 40× magnification. Micronuclei are indicated by yellow arrows; the white line represents a scale bar of 50 µm.

The antigenotoxic potential of *A. deliciosa* leaf extract against MMC-induced DNA damage in CHO-K1 cells was evaluated, with results presented in Figure 6. As expected, MMC treatment alone caused a significant increase in micronuclei frequency compared to the NC. Co-treatment with *A. deliciosa* extract significantly reduced MMC-induced micronuclei formation in a dose-dependent manner. Specifically, cells treated with 75 μg/mL and 150 μg/mL of the extract exhibited a significant decrease in micronuclei frequency relative to the MMC-positive control, with inhibition rates of approximately 40% and 60%, respectively. In contrast, the lowest concentration tested (37.5 μg/mL) did not confer significant protection. Representative fluorescence microscopy images illustrating the protective effects observed at higher extract concentration compared with MMCs are shown in Figure 7.

## 4. Discussion

Leaves of *A. deliciosa* constitute a promising yet underutilized agro-industrial by-product due to their bioactive composition. In this study, cytotoxicity assessment was conducted to support the interpretation of genotoxicity data. Since excessive cytotoxicity can lead to secondary cellular effects that interfere with genotoxic endpoints [25,26], concentration ranges were selected to maintain cell viability and metabolic activity, thereby ensuring robust and interpretable results. In this study, cell viability assays were performed to evaluate the cytotoxic effects of *A. deliciosa* leaf extract and to establish suitable concentrations for subsequent genotoxicity testing. The literature on the cytotoxicity of *A. deliciosa* leaves is limited, and no studies have specifically examined its effects on CHO-K1 cells. The ultrasound-assisted proanthocyanidin extracts of *A. deliciosa* leaves significantly reduced the viability of Caco-2 cells at concentrations above 25 µg/mL and HepG2 cells above 50 µg/mL after 48 h, as measured by the MTT assay [27]. Our study showed that the leaf extract exhibits moderate cytotoxicity (IC_50_ = 156 μg/mL in CHO-K1 cells), whereas in another study, fruit extracts were found to be non-cytotoxic up to 800 μg/mL in human lymphocyte cells [9]. In addition to differences in plant parts, it is important to note that these studies were conducted using different cellular models. Differences in cell type, species origin, and cellular sensitivity may contribute to the observed variation in cytotoxic responses [28]. Furthermore, this difference may also reflect variations in phytochemical composition between leaves and fruits.

Plant extracts are known to possess genotoxic or anti-mutagenic/anti-genotoxic activities [19,21]. Anti-genotoxicity is defined as the potential of a substance to mitigate genetic damage induced by a genotoxic agent. In the current study, we investigated the genotoxic and anti-genotoxic effects of *A. deliciosa* leaf extract against the known genotoxic agent MMC. The results showed that the selected concentrations of the *A. deliciosa* extract were not genotoxic on their own and effectively reduced the genetic damage caused by MMC. This suggests that compounds within the *A. deliciosa* extract are not genotoxic and possess an anti-genotoxic effect when co-administered with MMC. The observed protective effect with a significant reduction in micronuclei frequency in CHOK1 cells maybe attributable to the direct action of the extract’s compounds.

This study is the first to investigate the genotoxic and antigenotoxic potential of *A. deliciosa* leaf extract. Previous genotoxicity research has focused on other parts of the plant, particularly the fruit, which generally demonstrates protective, non-genotoxic effects. *A. deliciosa* fruit has been shown to modulate DNA damage and repair [10]. For example, *A. deliciosa* fruit juice exhibited a protective effect against the genotoxicity of heterocyclic aromatic amines in metabolically competent V79 cells, as determined by the comet assay [29]. Methanolic fruit extracts were also found to be non-genotoxic to human lymphocytes in Sister Chromatid Exchange and Chromosomal Aberration assays and exhibited radioprotective effects, reducing chromosomal aberrations after irradiation, likely due to their phenolic content and antioxidant activity [9]. In a human dietary intervention, regular consumption of golden kiwifruit protected lymphocytes from DNA oxidation and increased total antioxidant activity, particularly plasma vitamin C, while also influencing DNA repair pathways [30].

The anti-genotoxic effect observed in this study indicates that phytochemicals within *A. deliciosa* leaf extracts are non-genotoxic and can protect cells when co-administered with MMC. This protective effect, reflected by a significant reduction in micronuclei frequency in CHO-K1 cells, is likely due to the direct action of the extract’s bioactive metabolites. In this study, phytochemical analysis identified quercetin-3-O-rutinoside (rutin) as the most abundant metabolite, consistent with previous reports by [7], which found key phytochemicals in the aqueous leaf fraction, including quercitrin, rutin, procyanidins B and C, quinic acid, myricitrin, and triterpene acid-O-hexoside. Quercetin 3-O-rutinoside is a well-established antioxidant with a safety profile [31,32]. Previous studies have demonstrated the antigenotoxic potential of quercetin 3-O-rutinoside and quercetin in both in vitro and in vivo systems. For instance, quercetin 3-O-rutinoside and quercetin significantly reduced radiation-induced chromosomal damage in the bone marrow cells of mice exposed to gamma radiation [33]. The scavenging and antioxidant properties of these phytochemicals play a key role in conferring protection against gamma radiation. Previous studies have shown that quercetin-3-O-rutinoside, when combined with other phytochemicals such as podophyllotoxin and its glucoside derivative, enhances survival and protects radiosensitive hematopoietic and gastrointestinal tissues [34]. More recent research indicates that quercetin-3-O-rutinoside alone can effectively safeguard the hematopoietic and pulmonary systems in mice exposed to sublethal gamma radiation [35,36]. Additionally, it was demonstrated that quercetin-3-O-rutinoside protects the gastrointestinal tract of C57BL/6 mice from gamma radiation–induced damage by modulating antioxidant and inflammatory responses and reducing apoptosis in intestinal tissue [37]. The observed anti-genotoxic activity of *A. deliciosa* leaf extract in this study can be attributed to the presence of quercetin-3-O-rutinoside, which likely mediates this protective effect. While rutin is a well-documented antioxidant, the precise mechanism underlying its antigenotoxic effect in this model remains to be fully elucidated. It is possible that this protection involves free radical scavenging; however, other mechanisms, such as modulation of xenobiotic-metabolizing enzymes or stimulation of DNA repair pathways, may also contribute to the observed antigenotoxic effect. Further studies are needed to clarify the mechanisms by which *A. deliciosa* leaf extract exerts its antigenotoxic activity. While the current analysis of rutin’s antigenotoxic properties offers useful context, it is important to differentiate between different types of DNA damage. Much of the above-cited literature focuses on damage caused by ionizing radiation, which operates through mechanisms that differ from the interstrand cross-linking induced by mitomycin C (MMC). As a result, the biochemical pathways through which rutin protects against radiation-induced damage may not be directly applicable to the mechanisms involved in counteracting MMC-induced genotoxicity.

Another metabolite identified in the *A. deliciosa* leaf extract in this study was kaempferol-O-hexoside. Certain kaempferol glycosides, such as astragalin (kaempferol-3-O-glucoside), are well-recognized bioactive natural flavonoids with significant medicinal value. They have been reported to possess a range of pharmacological activities, including antioxidant, anti-inflammatory, anticancer, and neuroprotective effects [38,39]. Furthermore, recent findings indicate that kaempferol-3-O-glucoside and kaempferol-3-O-rutinoside offer protective effects against ethanol-induced damage, including genotoxicity, in the liver cells of Wistar rats [40].

Another metabolite identified in the *A. deliciosa* leaf extract in this study was (+)-catechin and (−)-epicatechin, along with several catechin derivatives, including (epi)catechin–(epi)catechin–(epi)catechin isomers. The findings from our study align with previous reports [11,12]. The genotoxic and antigenotoxic properties of catechin isolated from other plant sources, such as the bark of *Hamamelis virginiana* L., have been evaluated in HepG2 cells using the comet assay. Catechin, together with hamamelitannin and two proanthocyanidin fractions, induced only minimal DNA damage at concentrations up to 166 μg/mL. Importantly, pretreatment of HepG2 cells with catechin at 18 μg/mL reduced benzo[a]pyrene-induced DNA damage by 50% [41]. Similar dual effects have been reported for (-)-Epigallocatechin gallate (EGCG), in human lymphocytes, where 200 μM EGCG increased oxidative DNA damage, and concentrations above 30 μM induced DNA double-strand breaks in human lung and skin cells, whereas lower EGCG concentrations (0.01–10 μM) significantly reduced H_2_O_2_-induced DNA damage [42]. In Jurkat T-lymphocytes, EGCG likewise exhibited a biphasic response, inducing oxidative damage at concentrations above 100 μM while exerting protective effects at 10 μM [43].

The phytochemical characterization of *A. deliciosa* leaf extracts revealed the presence of low-polar pentacyclic triterpenoids, including oleanolic acid and ursolic acid. These compounds have been reported to significantly reduce micronucleus frequency induced by the clastogenic effects of doxorubicin in mouse peripheral blood and bone marrow cells [44]. Furthermore, ref. [45], demonstrated that ursolic acid inhibits aflatoxin B1-induced mutagenicity. Similarly, Guevara et al. observed a reduction in micronucleated polychromatic erythrocytes induced by mitomycin C in the bone marrow of Swiss mice, supporting the antimutagenic potential of ursolic acid. In addition, ursolic acid isolated from Uncaria sinensis (Oliv.) Havil exhibited a suppressive effect on the SOS-inducing activity of the mutagenic heterocyclic amine Trp-P-1 in the Salmonella typhimurium TA1535/pSK1002 umu test [46]. Overall, the antigenotoxic effects of *A. deliciosa* leaf extracts may be attributed, at least in part, to the presence of these triterpenoids, particularly oleanolic acid and ursolic acid.

## 5. Conclusions

In recent years, there has been increasing interest in exploring phytochemicals as natural alternatives to synthetic compounds commonly used in the food, pharmaceutical, and cosmetic industries. This study demonstrates that *A. deliciosa* leaf extract, an as agro-industrial byproduct, is a rich source of bioactive metabolites, particularly proanthocyanidins, flavonols such as quercetin-3-O-rutinoside, and triterpenoid saponins. The extract exhibited no genotoxicity in CHO-K1 cells and showed substantial antigenotoxic activity, reducing MMC-induced micronuclei by up to 60%. These protective effects are likely associated with the phytochemical constituents presented in the extract. While these findings suggest that the extract may represent a promising natural chemopreventive agent with a favorable safety profile, further validation through in vivo studies and comprehensive mechanistic investigations is required. Overall, *A. deliciosa* leaf extract represents a promising candidate for future health-promoting applications and supports the sustainable utilization of agricultural residues.

## Figures and Tables

**Table 1:** Metabolomics profile of methanol extract of *A. deliciosa* leaves.

Peak Number	Putative Identification	Elemental Formula	Class
1	Trihexose, chloride adduct	C_18_H_32_O_16_	Oligosaccharides
2	Tetrahexose chloride adduct	C_24_H_42_O_21_	Oligosaccharides
3	Dihexose, chloride adduct	C_12_H_22_O_11_	Oligosaccharides
4	Trihexose, formic acid adduct	C_18_H_32_O_16_	Oligosaccharides
5	Ascorbic acid hexoside	C_12_H_18_O_11_	Organic acids
6	Hexose derivative	C_11_H_22_O_9_	
7	Citric acid	C_6_H_8_O_7_	Organic acids
8	Pyridoxine hexoside	C_14_H_21_NO_8_	Pyridines
9	Gallic acid hexoside	C_13_H_16_O_10_	Gallic acid derivative
10	(Epi)gallocatechin-(epi)gallocatechin	C_30_H_26_O_14_	Proanthocyanidins
11	Dihydroxybenzoic acid glucoside	C_13_H_16_O_9_	Hydroxybenzoic acids
12	(Epi)gallocatechin-(epi)catechin	C_30_H_26_O_13_	Proanthocyanidins
13	Esculin	C_15_H_16_O_9_	Hydroxycoumarin
14	(Epi)catechin-(Epi) catechin isomer 1	C_30_H_26_O_12_	Proanthocyanidins
15	(Epi)gallocatechin-(epi)gallocatechin-(epi)catechin	C_45_H_38_O_20_	Proanthocyanidins
16	(Epi)gallocatechin-(epi)catechin)-(epi)gallocatechin-(epi)catechin	C_60_H_50_O_26_	Proanthocyanidins
17	Unidentified	C_21_H_24_O_11_	
18	(+)-Catechin	C_15_H_14_O_6_	Flavan-3-ols
19	(Epi) gallocatechin-(epi) catechin) -(epi) catechin isomer 1	C_45_H_38_O_19_	Proanthocyanidins
20	(Epi)catechin-(Epi) catechin isomer 2	C_30_H_26_O_12_	Proanthocyanidins
21	(Epi)catechin-(Epi) catechin isomer 3	C_30_H_26_O_12_	Proanthocyanidins
22	(Epi)catechin-(Epi) catechin-(epi) catechin isomer 1	C_45_H_38_O_18_	Proanthocyanidins
23	(Epi)gallocatechin-(epi) catechin-(epi) catechin isomer 2	C_45_H_38_O_19_	Proanthocyanidins
24	(−)-Epicatechin	C_15_H_14_O_6_	Flavan-3-ols
25	Unidentified	C_19_H_28_O_10_	
26	(Epi)catechin-(Epi) catechin-(epi) catechin isomer 2	C_45_H_38_O_18_	Proanthocyanidins
27	(Epi)catechin-(Epi)catechin-(epi)catechin-(epi)catechin	C_60_H_50_O_24_	Proanthocyanidins
28	Myricetin-O-deoxyhexosylhexoside	C_27_H_30_O_17_	Flavonols
29	Myricetin-3-O-glucoside	C_21_H_20_O_13_	Flavonols
30	Quercetin-3-O-rutinoside (Rutin)	C_27_H_30_O_16_	Flavonols
31	Myricetin-3-O-rhamnoside (Myricitrin)	C_21_H_20_O_12_	Flavonols
32	Quercetin-3-O-glucoside (Isoquercetin)	C_21_H_20_O_12_	Flavonols
33	Kaempferol-3-O-rutinoside (Nicotiflorin)	C_27_H_30_O_15_	Flavonols
34	Kaempferol-3-O-glucoside (Astragalin)	C_21_H_20_O_11_	Flavonols
35	Kaempferol-3-O-rhamnoside (Kaempferin)	C_21_H_20_O_10_	Flavonols
36	Phlorizin, formic acid adduct	C_21_H_24_O_10_	Chalcones
37	Pentahydroxyurs-12-en-28-oic acid hexoside or structural isomer	C_36_H_58_O_12_	Ursane and Taraxastane triterpenoids
38	Pentahydroxyursa-12,20(30)-dien-28-oic acid hexoside or structural isomer	C_36_H_56_O_12_	Ursane and Taraxastane triterpenoids
39	Tetrahydroxyurs-12-en-28-oic acid hexoside or structural isomer	C_36_H_58_O_11_	Ursane and Taraxastane triterpenoids
40	Trihydroxy-12,20(30)-ursadien-28-oic acid (Actinidic acid) acetylhexoside or structural isomer	C_38_H_60_O_12_	Ursane and Taraxastane triterpenoids
41	Pentahydroxyurs-12-en-28-oic acid or a structural isomer	C_30_H_48_O_7_	Ursane and Taraxastane triterpenoids
42	Unidentified	C_52_H_80_O_22_	
43	Unidentified	C_52_H_80_O_22_	
44	Unidentified	C_54_H_84_O_22_	
45	Tetrahydroxyurs-12-en-28-oic acid or a structural isomer	C_30_H_48_O_6_	Ursane and Taraxastane triterpenoids
46	Trihydroxy-12,20(30)-ursadien-28-oic acid (Actinidic acid) or structural isomer 1	C_30_H_46_O_5_	Ursane and Taraxastane triterpenoids
47	Coumaroyl tetrahydroxyurs-12-en-28-oic acid or structural isomer 1	C_39_H_54_O_8_	Ursane and Taraxastane triterpenoids
48	Trihydroxyolean-12-en-28-oic acid or a structural isomer	C_30_H_48_O_5_	Oleanane triterpenoids
49	Tetrahydroxyursa-12, 20(30)-dien-28-oic acid or structural isomer	C_30_H_46_O_6_	Ursane and Taraxastane triterpenoids
50	Trihydroxyurs-12-en-28-oic acid or structural isomer 1	C_30_H_48_O_5_	Ursane and Taraxastane triterpenoids
51	Trihydroxyurs-12-en-28-oic acid or structural isomer 2	C_30_H_48_O_5_	Ursane and Taraxastane triterpenoids
52	Coumaroyl tetrahydroxyurs-12-en-28-oic acid or structural isomer 2	C_39_H_54_O_8_	Ursane and Taraxastane triterpenoids
53	Digalactosyl monoacylglycerol (C18:3)	C_33_H_56_O_14_	Glycolipids
54	Coumaroyl tetrahydroxyurs-12-en-28-oic acid or structural isomer 3	C_39_H_54_O_8_	Ursane and Taraxastane triterpenoids
55	Trihydroxyurs-12-en-28-oic acid or structural isomer 3	C_30_H_48_O_5_	Ursane and Taraxastane triterpenoids
56	Trihydroxyurs-12-en-28-oic acid or structural isomer 4	C_30_H_48_O_5_	Ursane and Taraxastane triterpenoids
57	Monogalactosyl monoacylglycerol (C18:3)	C_30_H_46_O_9_	Glycolipids
58	Trihydroxy-12,20(30)-ursadien-28-oic acid (Actinidic acid) or structural isomer 2	C_30_H_46_O_5_	Ursane and Taraxastane triterpenoids
59	Coumaroyl trihydroxyurs-12-en-28-oic acid or structural isomer 1	C_39_H_54_O_7_	Ursane and Taraxastane triterpenoids
60	Dihydroxyolean-12-en-28-oic acid or a structural isomer	C_30_H_48_O_4_	Oleanane triterpenoids
61	Dihydroxyurs-12-en-28-oic acid or a structural isomer	C_30_H_48_O_4_	Ursane and Taraxastane triterpenoids
62	Maslinic acid	C_30_H_48_O_4_	Oleanane triterpenoids
63	Corosolic acid	C_30_H_48_O_4_	Ursane and Taraxastane triterpenoids
64	Coumaroyl trihydroxyurs-12-en-28-oic acid or structural isomer 2	C_39_H_54_O_7_	Ursane and Taraxastane triterpenoids
65	Coumaroyl dihydroxyurs-12-en-28-oic acid or structural isomer 1	C_39_H_54_O_6_	Ursane and Taraxastane triterpenoids
66	Coumaroyl dihydroxyurs-12-en-28-oic acid or structural isomer 2	C_39_H_54_O_6_	Ursane and Taraxastane triterpenoids
67	Violaxanthin or a structural isomer	C_40_H_56_O_4_	Carotenoids
68	Oleanolic acid	C_30_H_48_O_3_	Oleanane triterpenoids
69	Ursolic acid	C_30_H_48_O_3_	Ursane and Taraxastane triterpenoids

## Data Availability

The original contributions presented in this study are included in the article/Supplementary Materials. Further inquiries can be directed to the corresponding author.

## Supplementary Materials

The following supporting information can be downloaded at: https://www.mdpi.com/article/10.3390/toxics14040324/s1. Table S1: The tentatively assigned compound identities in *A. deliciosa* Leaves and the corresponding chromatographic peaks. Additional details, including retention times, experimental *m*/*z* values, molecular formulas, and diagnostic fragment ions, are also reported in Supplementary Table S1.
